# Mandibular Lateral Displacement in Growing Patients: Etiopathogenesis, Prophylaxis, and Early Treatment—A Literature Review

**DOI:** 10.3390/jcm14228090

**Published:** 2025-11-14

**Authors:** Karolina Kowalska, Agnieszka Machorowska-Pieniążek

**Affiliations:** Department of Orthodontics, Faculty of Medical Sciences in Zabrze, Medical University of Silesia, 40-055 Katowice, Poland

**Keywords:** mandibular lateral displacement, facial asymmetry, crossbite, orthodontic prophylaxis, functional treatment

## Abstract

**Background**: Mandibular lateral displacement is a common functional occlusal disorder that may occur independently or in conjunction with other types of malocclusion. Substantial evidence indicates that in growing patients, mandibular lateral displacement can have a significant negative impact on the development of both the morphology and function of the stomatognathic system. Early diagnosis and intervention are therefore crucial to preventing a range of developmental abnormalities, including skeletal and soft tissue asymmetries, temporomandibular joint dysfunction, and pathological tooth wear. **Aim**: The aim of this study is to analyze and systematize current scientific perspectives on the etiopathogenesis of mandibular lateral displacement, as well as approaches to its prevention and early treatment. **Methods**: A comprehensive review of the literature published between 1980 and 2025 was conducted. Sources were identified through searches in the PubMed, Google Scholar, Elsevier, Web of Science, and SUM Library databases. **Results**: Mandibular lateral displacement necessitates early diagnosis and timely initiation of orthodontic treatment. The majority of studies addressing the treatment of mandibular lateral displacement are limited to case reports. **Conclusions**: Further well-designed, longitudinal studies are needed to establish standardized diagnostic criteria and evidence-based treatment protocols for early management of mandibular lateral displacement in growing patients.

## 1. Introduction

Mandibular lateral displacement (MLD) belongs to a group of transverse maxillofacial disorders characterized by asymmetry of the lower face. Extraorally, it presents as a lateral displacement of the chin relative to the facial midline, asymmetry of the lips and a deepened nasolabial fold. Intraorally, clinical findings include anterior or posterior crossbite, as well as disturbances in mandibular abduction and adduction. Functional testing typically yields a positive or intermediate result [1,2]. In all cases, MLD essentially represents a three-dimensional rotation of the mandible around the vertical axis, in which the mandibular head on the side opposite the deviation is displaced forward, downward, and medially together with the articular disc [3,4].

The reported prevalence of MLD varies across studies. According to Kuting et al., MLD occurs in 8.4% of children with primary dentition and 7.2% of those with mixed dentition [5]. Other authors, however, describe a substantially higher prevalence, considering MLD a common condition [6,7,8,9,10]. Severt et al. reported that 74% of patients demonstrate facial asymmetry associated with transverse mandibular displacement [6], with 80–90% of cases affecting the left side [6,7,8,9,10]. The main intraoral manifestation of MLD is the presence of a crossbite [11]. In 80–97% of patients with posterior crossbite, whether in the primary or mixed dentition, lateral mandibular displacement is observed [5,11,12,13,14]. Most often, as a result of maxillary constriction, occlusion of the dental arches leads to a forced lateral displacement of the mandible, resulting in the development of functional posterior crossbite (FPXB) in the posterior teeth [14]. According to several authors, FPXB arises from symmetrical maxillary narrowing, which causes occlusal interferences in the lateral segments. Due to muscular instability, the mandible deviates to one side, accompanied by an altered position of the mandibular condyles within the articular fossae. This condition is associated with both facial asymmetry and chin deviation [12,13,14]. Many researchers also emphasize the coexistence of MLD with temporomandibular disorders (TMDs) [15,16,17]. In a study by Fushima et al., 65% of patients with MLD were diagnosed with TMD, with 84.6% of symptoms occurring in the joint on the side of displacement, compared to only 23.1% on the unaffected side [17].

The relationship between craniofacial morphology and the prevalence of MLD has also been investigated. The findings suggest that MLD occurs more frequently in patients with Class III malocclusion than in those with Class II malocclusion [18,19,20,21]. Research on mandibular lateral displacement (MLD) is of high clinical and scientific relevance. Early identification and management of this condition are essential for maintaining proper growth and harmonious development of the stomatognathic system. Undiagnosed or untreated MLD during the growth period can result in progressive facial asymmetry, temporomandibular joint dysfunction, altered mandibular kinematics, and malocclusion. Over time, these disturbances may lead to permanent skeletal deformities that are difficult to correct in adulthood and often require combined orthodontic–surgical intervention.

Despite numerous clinical reports, there is still a lack of consensus regarding the multifactorial origin of mandibular displacement and the most effective preventive and early therapeutic approaches. The aim of this systematic review was to summarize the current state of knowledge regarding the diagnosis, etiopathogenesis, and principles of early treatment in growing patients with mandibular lateral displacement.

## 2. Materials and Methods

**Figure 1:**
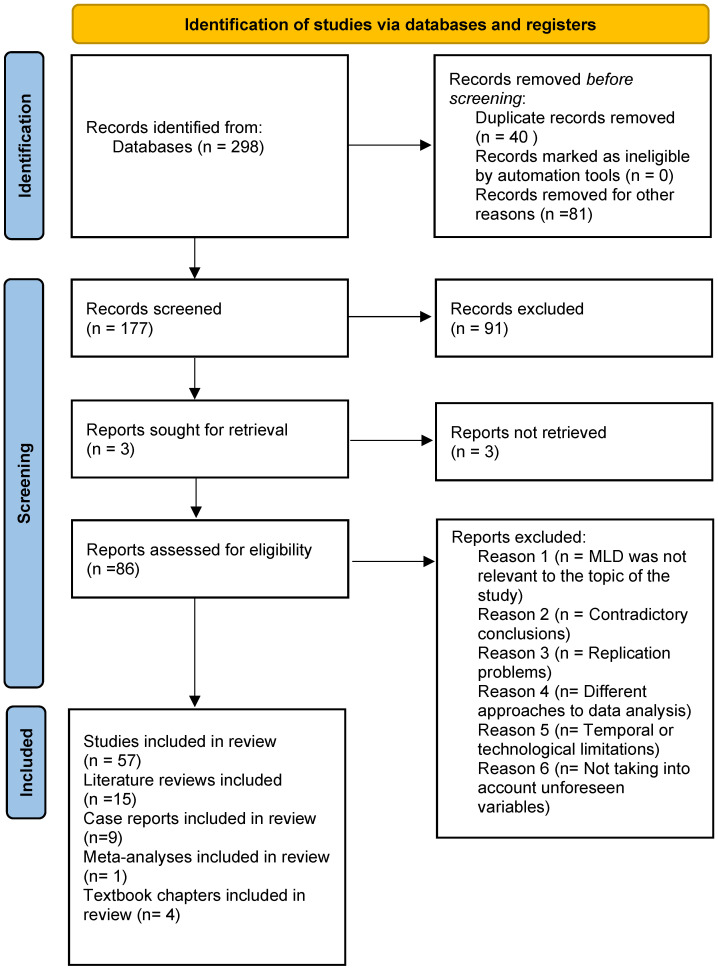
Flowchart of the study selection process.

This review was designed to be transparent and complies with PRISMA guidelines (Figure 1). In the initial phase, 298 publications were identified. After verification, 86 studies were ultimately included in the analysis. Publications lacking a clear definition of mandibular lateral displacement, articles in which MLD was only mentioned as a disorder but was not relevant to the topic of the study, or presenting, in the authors’ opinion, contradictory conclusions, replication problems, different approaches to data analysis, or did not take into account unforeseen variables, or presented temporal or technological limitations were excluded.

A comprehensive literature review covering the years 1980 to 2025 was conducted in the Google Scholar, Web of Science, PubMed, Elsevier, and SUM Library databases, including publications in Polish, English, and German. Additionally, earlier articles considered relevant by the authors were also taken into account. The review encompassed randomized controlled trials, systematic reviews, meta-analyses, observational studies, cross-sectional studies case reports, and textbook chapters. The following keywords were applied in the search process: Mandibular Lateral Displacement, Mandibular Lateral Shift, Mandibular Lateral Deviation, MLD, Functional Shift of the Mandible.

The articles were evaluated using the CASP (Critical Appraisal Skills Programme) scale. The results of the analysis indicated that 17.86% of the works were of moderate quality and 82.13% were of high quality according to the adopted evaluation criteria. We recommend caution in interpreting the results of the analysed publications and hope that future studies will have higher methodological standards. Considering the above, we assess the study as positive in terms of exploring the subject of lateral mandibular displacement.

## 3. Results and Discussion

The diagnosis of mandibular lateral displacement (MLD) is primarily based on the observation of facial asymmetry, typically manifested by lateral deviation of the chin in maximum intercuspation. Facial symmetry is assessed using standardized extraoral orthodontic photographs taken in the “norma frontalis,” “norma basalis,” and “norma zygomatica” projections. This type of analysis allows for a rapid, precise, and non-invasive evaluation of the facial regions affected by asymmetry and facilitates the detection of additional features indicative of multidimensional facial asymmetry [22]. Intraoral examination reveals misalignment of the midlines of the dental arches, most frequently accompanied by posterior or anterior crossbite [23]. Confirmation of MLD requires a functional test, which consists of guiding mandibular movements and repositioning the mandible so that the midline of the lower arch coincides with the midline of the upper arch and the facial midline. The ability to achieve this repositioning, along with improvement in facial symmetry and occlusal relationships, indicates a functional etiology of the condition [2,24,25,26,27]. A functional test result is considered intermediate when mandibular repositioning is possible but leads to deterioration of facial symmetry or occlusal conditions worsen [2].

### 3.1. Morphological Characteristics Associated with Mandibular Lateral Deviation (MLD)

As a consequence of lateral displacement of the mandible, asymmetry arises within the occlusal plane, the maxillary alveolar process and teeth, the alveolar part, body and ramus of the mandible, as well as the cranial base. The most frequently reported morphological alterations include:

- upward inclination of the occlusal plane toward the side of mandibular displacement [28];
- a steeper posterior segment of the occlusal plane on the displacement side compared to the contralateral side [28];
- shortening of the maxillary teeth and the corresponding alveolar process on the side of mandibular displacement [29,30];
- inclination of the occlusal plane resulting from three-dimensional mandibular displacement toward the side characterized by a smaller vertical dimension [30];
- increased mandibular body length on the side of displacement [29];
- increased effective mandibular length (Condylion–Pogonion) and ramus length (Condylion–Gonion) on the contralateral side relative to the mandibular displacement [29];
- reduction in the length, height, and width of the mandibular condylar head on the side of displacement [19,31,32,33].

### 3.2. Functional Characteristics Associated with Mandibular Lateral Displacement (MLD)

Functional disturbances accompanying mandibular lateral displacement (MLD) predominantly involve neuromuscular asymmetry, altered mandibular kinematics, and compensatory temporomandibular joint (TMJ) adaptations. The most frequently documented functional manifestations include:

- Neuromuscular imbalance and asymmetrical muscle function — reduced tone of the masseter muscle on the deviated side compared to the opposite side, accompanied by increased tone of the pterygoid muscles [34,35]. The particularly noteworthy study was conducted by Hao Guan et al. on growing rats with experimentally induced lateral functional mandibular displacement [36]. The induced displacement resulted in atrophy of the masseter and temporalis muscles on the deviated side and hypertrophy on the contralateral side. Moreover, asymmetric decomposition of muscle fiber types and cross-sectional area was observed. The overall body weight of the animals remained stable, indicating an unimpaired ability to feed. The researchers demonstrated that mechanical stimulation affected the expression of regulatory proteins involved in the growth of the temporalis and masseter muscles, causing increased expression of GDF-8 (Growth Differentiation Factor-8) and decreased levels of IGF-1 (Insulin-Like Growth Factor-1) on the deviated side, with opposite effects in the contralateral muscles. After elimination of the mechanical disturbance, the muscles regained their original structure and function, identical to the pre-experimental state. According to the authors, this finding indicates that at the early stage, changes induced by functional mandibular lateral displacement are completely reversible. According to the authors, this finding indicates that at the early stage, changes induced by functional mandibular lateral displacement are completely reversible [36];
- Compensatory displacement of the mandibular condyles within the articular fossae, resulting from anterior translation or tilting of the condylar head on the ipsilateral side [37,38]. This phenomenon was demonstrated by Nerder et al. following the determination of the therapeutic mandibular position using a bite splint [37];
- Asymmetrical masticatory patterns-due to disproportionate activity of the chewing muscles on both sides [29,39]. Nakaminami et al. demonstrated that individuals with MLD exhibit shorter masticatory trajectories with predominance of horizontal movements [40];
- Temporomandibular joint (TMJ) dysfunction, manifested as abnormalities during mandibular opening and closing. Mandibular displacement occurs not only at the terminal phase of the movement but also throughout its execution [37]. Research by Qi Chen et al. demonstrated the occurrence of acoustic TMJ symptoms (clicking sounds) in MLD patients during the initial and terminal phases of mandibular opening. This observation may suggest concurrent abnormalities in the articular disc [26].

### 3.3. Etiology of Mandibular Lateral Displacement (MLD)

Mandibular lateral displacement represents a multifactorial condition arising from various local and systemic factors.

Premature tooth contact caused by both dental-occlusal abnormalities and premature tooth loss is considered the most common pathological factor causing MLD [25,41,42,43].

Abnormal dental upper arch width can cause asymmetrical neuromuscular impulses, resulting in pathological mandibular guidance (inappropriate abduction and adduction) [44].

Skeletal asymmetries are one of the causes of mandibular displacement. In adults, the prevalence of asymmetry is reported to be 48%. They may involve excessive or insufficient growth of one or more bones of the facial part of the skull [34,45]. In some cases, mandibular displacement occurs as a compensatory response to asymmetries in the maxilla, occlusal plane inclination, or dental arch morphology.

Torticollis (TC) is defined as a perinatal deformity associated with unilateral imbalance of the sternocleidomastoid muscle (SCM)—including its shortening, tension, and dysfunction. This leads to neck straightening disorders and more frequent turning of the head to one side. Characteristic features include: forward displacement of the ear, flattening of the occiput on the opposite side to the torticollis, asymmetry of the face and eyes, and displacement of the mandible. According to Cheng et al., 62.2% of patients with MLD have a history of complicated delivery, forceps delivery, or cesarean section [46]. Torticollis is associated with changes in the inclination of the mandible, which can lead to shortening of the mandibular ramus on the side of the torticollis. TC is a common cause of MLD. In mild cases, malocclusion may be the first noticeable clinical symptom of this disorder [29,31,47,48,49,50].

Scoliosis is an idiopathic, progressive orthopedic disease characterized by abnormal posture. It is characterized by a lateral deviation of the spine from the midline of the body [51,52,53]. Numerous authors have emphasized a higher incidence of malocclusion [54], temporomandibular joint disorders [55] in patients with scoliosis. Occlusal and non-occlusal parafunctions have been observed in people with scoliosis compared to healthy individuals [56]. There are reports of the coexistence of MLD with scoliosis and symmetry disorders of the upper and lower dental arches. Cabrera-Domínguez et al. state that scoliosis in the cervical and thoracic spine can cause displacement of the midline of the teeth and displacement of the mandible in the same direction, and in the lumbar spine in the opposite direction [57]. However, the conclusions of scientific publications are not clear regarding the relationship between MLD and scoliosis. No relationship between the direction of mandibular displacement and the direction of scoliosis has been noted [58,59,60,61,62]. The available literature presents the effectiveness of physiotherapy in supporting the treatment of malocclusion [53,63,64,65,66].

Plagiocephaly is a condition associated with perinatal trauma or intrauterine abnormalities of the fetus. It is exacerbated by abnormal head position, especially during sleep (lying on the back). It is essentially a deformation of the base of the skull. Head tilt and limited neck mobility are consequences of not treating this disorder. It is believed that torticollis can lead to plagiocephaly. Research by Sasaki et al. has shown that MLD in this case is associated with an increase in the volume of the temporal muscle on the side of the disorder and tilting of the head, along with the mandible, to the opposite side [67]. The extent of MLD and surrounding soft tissue disorders depends on the degree of jaw tilt [31,67,68,69].

Mandibular skeletal asymmetry. According to Ishizaki et al. and Kim et al., skeletal asymmetry of the mandible can lead to the development of MLD in the mechanism of unilateral vertical occlusal compensation. According to the authors, bone asymmetry is compensated by unilateral intrusion of the molars. This condition may lead to a unilateral reduction in occlusal height, transverse inclination of the occlusal plane, and as a result, lateral displacement of the mandible [28,69].

### 3.4. Pathogenesis of Mandibular Lateral Displacement (MLD)

**Figure 2:**
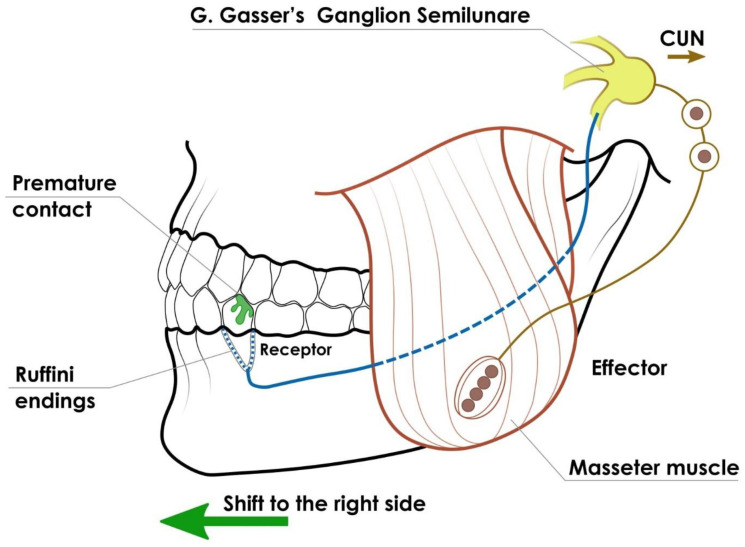
Schematic illustration of the neuromuscular reflex.

The main elements of the neuromuscular basis of MLD are activity at the level of involuntary reflexes stimulated by receptors (periodontium, TMJ, masticatory muscles proprioceptive organs), nerve impulse conduction (maxillary and mandibular nerves), and the response of effector organs (muscles connected to the mandible). Premature occlusal contacts occurring on one side of the dental arch play an important role in the development of MLD Figure 2. During the deciduous and mixed dentition periods, they are most often caused by caries, ectopic tooth eruption, dental abnormalities, and fillings that are not adapted to the bite. These irregularities may result in the mandible bypassing articulatory obstacles and being displaced during maximum intercuspation. In order to achieve occlusion that protects the teeth, muscles, and TMJ, muscles on one side contract with greater amplitude, which over time becomes a habitual neuromuscular pattern [70,71]. Ishizaki et al. identified differences in the height of occlusal points on the molars on the displaced side and the unaffected side as the main cause of MLD. The author also points to a mechanism of MLD associated with asymmetrical mandibular guidance, causing unilateral chewing. Uneven occlusal loading prevents symmetrical eruption of teeth on both sides, contributing to the perpetuation of bone asymmetry [28].

### 3.5. Prevention and Early Treatment of Mandibular Lateral Displacement (MLD)

1. Grinding of deciduous tooth cusps. After examination with articulating paper, the procedure is performed with a water-cooled diamond bur. In MLD, the palatal cusps of the upper deciduous molars and the buccal cusps of the lower deciduous molars are most often ground on the affected side. Premature contacts should be assessed repeatedly before the procedure and ground gradually. A positive therapeutic effect after selective grinding of premature contacts is reported in 27–64% of cases [33,72,73,74].
2. Myotherapy. After eliminating premature dental contacts, patients with lateral functional displacement of the mandible are recommended to perform certain exercises. Some of them consist of maximum movements of the mandible in the opposite direction to the disorder and return to the starting position (independent or forced movement with the hand) [75]. In addition, exercises are introduced to prevent mandibular displacement during adduction by stabilizing it in place. The occurrence of dysfunctions related to, among other things, mandibular movements, chewing, and swallowing should be assessed individually. In the event of abnormalities, myofunctional therapy and re-education should be introduced to eliminate these dysfunctions. The exercises should lead to the mandible reaching a resting position in the correct relationship with the midline of the face [26,33,73,74].
3. Onlays. Several authors have described the use of composite onlays to treat crossbite accompanied by MLD. They are designed to unblock the mandible, inhibit forced lateral movement, and enable proper transverse development of the maxilla by raising the bite [54]. Neto et al. describe a method combining selective grinding of deciduous tooth cusps with the creation of composite occlusal planes to achieve centric occlusion, proper mandibular guidance, and optimal TMJ function. Their use was also intended to ensure bite plane alignment [76]. Tuomo Kantomaa conducted studies on children with crossbite during the deciduous dentition period. He performed composite guiding surfaces on the buccal surface of the upper canine and first or second deciduous molar on the side of the disorder, in combination with grinding premature contacts on deciduous teeth. In most patients, the crossbite was corrected after one month [77]. Unilateral crossbite caused by MLD should be treated during the deciduous dentition stage [78].
4. Maxillary expansion. In cases where corrective grinding is insufficient, maxillary expansion is often performed first using rapid maxillary expansion (RME) or slow maxillary expansion (SME) [11,79]. Zineb Safi-Eddine et al. describe the frequent need to expand the upper dental arch during MLD treatment [80]. For this purpose, David B. Kennedy et al. point to the possibility of using appliances such as the W-arch, Quad Helix, Expander, Haas, or hyrax screw appliance in the deciduous and early mixed dentition to expand the maxilla. In early permanent dentition, Haas-type palatal expansion appliances with a hyrax or Superscrew are used [11]. In addition, Biega et al. present the effectiveness of Invisalign First aligners in the treatment of MLD [81].
5. Functional appliances. In the early treatment of MLD, functional appliances are used to influence the teeth, soft tissues, TMJ, and bone tissue. These appliances are made in disocclusion and the most correct position of the mandible in relation to the maxilla, taking into account the sagittal and orbital planes, after obtaining a positive functional test result. Szpinda-Barczyńsa et al. describe the effects of MLD therapy accompanying other malocclusions [82]. Case studies present the effectiveness of MLD treatment using the Klammt functional appliance, which can be used to widen the dental arches [83,84]. The U-bugel activator is also mentioned, which consists of two acrylic plates with occlusal surfaces for the teeth in the lateral sections, cut along the occlusal plane. Its wire elements include two lip arches and loops connecting the upper and lower acrylic plates in the shape of the letter U, which are turned upwards with their convexities. Activation of these loops enables compensatory displacement of the mandible [85]. Removable single-jaw appliances with an acrylic plate are most often used to correct the transverse dimension of the arches, using one or more expansion screws [86].

### 3.6. Authors’ Perspective

- In the author’s clinical practice, mandibular lateral displacement is a relatively frequent finding, particularly during early stages of craniofacial development. There are no current epidemiological data on its prevalence in the general Polish population or among orthodontic patients
- Early orthodontic intervention is recommended as soon as MLD is diagnosed. In patients with functional mandibular lateral displacement (MLD), the primary therapeutic objective is the elimination of occlusal interferences that prevent symmetrical positioning of the mandible. For this purpose, we use selective grinding of primary teeth, onlays, prosthetic restorations, as well as removable and fixed intraoral and extraoral appliances, taking into account individual indications.
- The treatment of mandibular lateral displacement (MLD) is conducted by an interdisciplinary team comprising an orthodontist, prosthodontist, physiotherapist, and speech therapist.
- In cases of severe morphological MLD, orthodontic treatment alone is often insufficient and requires orthognathic therapy.

## 4. Conclusions

The findings of the literature review revealed that the majority of studies addressing the treatment of mandibular lateral displacement are limited to case reports. Furthermore, the review identified a significant gap in the current body of literature, particularly concerning the effectiveness of early treatment strategies for mandibular lateral displacement. It did not provide conclusive scientific evidence regarding specific conditions under which functional mandibular lateral displacement (MLD) can be fully reversible. There is a need to establish standardized diagnostic criteria and evidence-based treatment protocols for early management of mandibular lateral displacement in growing patients. Despite these limitations, the following clinical recommendations can be made based on the literature review:

1. During the deciduous and mixed dentition stages, early diagnosis of the occlusal asymmetry, identification of its etiology, and initiation of orthodontic intervention are essential.
2. The diagnosis and management of mandibular lateral displacement should adopt a multidisciplinary, team-based approach.

Further well-designed longitudinal studies on mandibular lateral displacement (MLD) should focus on the following areas:

1. Standardization of Diagnostic Criteria—Development of clear, evidence-based criteria to differentiate functional from morphological mandibular lateral displacement, integrating clinical, radiological, and functional assessments.
2. Comparative Treatment Studies—Longitudinal studies comparing different early intervention strategies in children and adolescents to determine the most effective approaches for correcting MLD.
3. Reversibility and Timing—Assessment of the relationship between intervention timing and MLD reversibility.
4. Clinical Guidelines and Protocols—Establishment of evidence-based protocols for early detection, prevention, and treatment of MLD.

## Data Availability

No new data were created or analyzed in this study. Data sharing is not applicable to this article.
